# User-centered design of graphical user interfaces for telemedical robotic systems: development and usability study

**DOI:** 10.1007/s11548-026-03701-4

**Published:** 2026-06-15

**Authors:** Sven Kolb, Marie Kautt, Lars Wagner, Simon Saurbier, Sven Matthiesen, Dirk Wilhelm, Carolin Müller

**Affiliations:** 1 TUM University Hospital, Research Group MITI, School of Medicine and Health, Technical University of Munich, Munich, Germany; 2 Munich Institute of Robotics and Machine Intelligence (MIRMI), Technical University of Munich, Munich, Germany; 3 Institute of Product Engineering (IPEK), Karlsruhe Institute of Technology (KIT), Karlsruhe, Germany; 4 TUM University Hospital, Department of Surgery, School of Medicine and Health, Technical University of Munich, Munich, Germany; 5 TUM University Hospital, Department of Trauma Surgery, School of Medicine and Health, Technical University of Munich, Munich, Germany

**Keywords:** User-centered design, Telemedical robotics, Usability, Graphical user interface

## Abstract

**Purpose:**

We explore an approach to develop telemedical Graphical User Interfaces (GUIs) using User-centered Design (UCD) methods. In contrast with prior work that emphasizes system integration, we center on the GUI as the clinician’s primary interface.

**Methods:**

In a user-centered, two-stage process, we developed a modular and generalized GUI architecture and adapted it to a Robotic Ultrasound System (RUSS) and a robotic Tension Pneumothorax (tPTX) system. The GUIs were iteratively co-designed and evaluated with physicians using mixed methods (System Usability Scale (SUS), end-user-adapted heuristics, eye-tracking).

**Results:**

The results of the study confirm the benefits of UCD methods for the development of GUIs for telemedical robotic systems. Repeated exposure increased efficiency across tasks and scenarios, indicating the usability (training effect) and consistency (cross-scenario learning transfer) of a modular GUI architecture. The mixed-methods design provided complementary insights via triangulation, helped to analyze outliers, and revealed additional design issues not captured by one method alone.

**Conclusion:**

A user-centered GUI development process with multiple evaluation rounds can improve the usability of telemedical robotic systems for medical professionals. Our findings show that a modular architecture can facilitate the transfer of training effects across implementations and clinical use cases by preserving high-level structures and interaction concepts.

**Supplementary Information:**

The online version contains supplementary material available at https://doi.org/10.1007/s11548-026-03701-4.

## Introduction

The core principles of UCD (focus on end-users and their tasks, collect empirical data through user feedback, and apply iterative design cycles) have been shown to lead to improved usability, which is associated with numerous benefits such as increased productivity, reduced error rates, and higher acceptance levels [1, 2]. Despite an increasing focus on usability and user experience in medical device development, approaches such as UCD are still underrepresented in scientific work [3, 4]. Indeed, GUI development in general has so far played a limited role in many telemedical robotic systems. To date, such systems have primarily been employed in operating rooms or within the same hospital in the form of Teleoperated Surgical Robotic Systems (TSRSs) [5, 6]. Unlike intra-hospital robotic systems, telemedical robotic systems such as RUSS can enable the provision of healthcare over long distances, with physicians and patients typically located at different sites. Such systems often combine multimodal sensing, imaging, and teleoperation capabilities. Their GUIs must integrate these functionalities and are therefore central to ensuring high-quality treatment. Apart from a few initial commercial solutions, these systems are largely still in the research phase, with projects like ProteCT or ReMeDi demonstrating how telemedical GUIs can be designed to provide multimodal information [7–10].

## Related work

### UCD in telemedicine

Since the early 2000s, several authors have proposed general UCD frameworks for the healthcare sector. Johnson et al. outlined a structured approach for improving the usability of clinical interfaces [11]. Patel and Kushniruk highlighted perception- and working memory-oriented design principles such as progressive disclosure, coherent layout hierarchies, and context-sensitive alerts [12]. More recent studies point out the importance of avoiding cognitive overload, suggesting modular or dynamic GUIs [13, 14]. The benefits of modularity are also discussed in other domains of digital health, such as for Health Information Systems (HISs) [15]. Beyond generic guidelines, domain-specific requirement catalogs have been proposed for medical teleoperation systems. These include consistent control paradigms, low latency, robust visual/auditory feedback, safety-critical status displays, role-based permissions/workflows, and configurable information density as key GUI properties [16]. Clinical evaluations in time-critical scenarios likewise emphasize that GUI design and workflow alignment are decisive for diagnostic quality [7]. Methodological reviews summarize suitable evaluation approaches, including heuristics, think-aloud protocols, and performance metrics [17]. Complementary strategies combine theoretical UCD approaches with empirical case studies or rely on high-fidelity simulation laboratories to examine workflow and team interaction [18, 19].

### Open challenges

Over the past two decades, a considerable number of telemedical robotic systems have been developed and tested in feasibility and pilot studies [4, 20]. These efforts demonstrate the technological potential of robotic telemedicine and have led to a variety of prototypes, research platforms, and commercial systems. However, the primary focus of these developments has often been on technical performance and functional demonstration, rather than on the safe, efficient and intuitive interaction between physicians and telemedical robotic systems. We have identified several prominent drawbacks in existing work:

- **Dominance of feasibility studies and technical demonstrations:** A large proportion of existing research remains at the level of feasibility or proof-of-concept studies (e.g. [21–23]). These projects often succeed in demonstrating technical viability but rarely consider usability or workflow integration.
- **Fragmented GUIs:** Existing telemedical systems often rely on multiple, disconnected GUIs for ultrasound imaging, robotic control, and videoconferencing [22, 24–27]. This fragmentation increases physicians’ cognitive load and complicates their workflows [12].
- **Limited integration of UCD:** Despite evidence that UCD improves system acceptance in healthcare, telemedical robotics research still rarely applies these principles consistently [10, 28–30]. Many projects continue to rely on training physicians to adapt to the system, rather than adapting the system to meet physicians’ needs [3, 4, 31].
- **Insufficient evaluation methodologies:** Many telemedical robotic studies limit their evaluation to subjective feedback (questionnaires/interviews), although previous reviews have emphasized that relying on a single method, either subjective or objective, is insufficient to capture the complexity of user interaction with medical devices [32, 33]. Mixed-methods approaches that systematically combine both perspectives have been shown to provide more valid and comprehensive insights [26, 33–37]. In telemedical robotics, however, such approaches remain rare.
- **Heuristic evaluation in specialized medical domains:** Traditionally, heuristic evaluations are conducted by usability experts to ensure methodological rigor and comparability [38]. However, this can limit the ability to fully capture the specific needs and workflows of specialized medical practitioners. Several studies emphasize that in specialized domains such as healthcare, end-users should be directly involved in heuristic assessments, as they are best positioned to identify workflow-specific challenges and safety-relevant issues [39–41]. To date, however, most telemedical GUI studies have relied on expert-driven evaluations, neglecting end-user-focused heuristic validation. This issue can also be seen in other medical domains, such as HISs for radiology [42].

In this work, we demonstrate the user-centered development of a unified, modular GUI architecture that enables a safe, ergonomic, and clinically applicable workflow. While technical functionalities such as robotic ultrasound, palpation, or remote auscultation have already been demonstrated in prior studies, their integration into a coherent, user-centered system still remains mostly unexplored. Furthermore, we introduce functional implementations to address both diagnostic and interventional scenarios. Beyond the integration of telemedical robotic functions into a unified GUI, we also contribute methodologically. While heuristic evaluation is usually conducted by usability experts, this work reformulates Nielsen’s heuristics into a SUS-aligned questionnaire [43–45]. This enables physicians to evaluate systems from a clinical end-user perspective. Finally, we combine these subjective usability ratings with objective interaction data from eye-tracking, which provides complementary insights into usability and reveals additional design issues not captured by SUS or heuristics alone.

## Methods

### UCD process

We implemented a two-stage UCD process, consisting of two iterative cycles with four main steps each: analysis, requirement derivation, prototype design, and systematic evaluation. In the first cycle, a static prototype was developed and evaluated by physicians, using standardized questionnaires and heuristic criteria, to identify general strengths and weaknesses of layout and structure and capture further design wishes. In the second cycle, insights from this evaluation were analyzed and integrated into the development of two functional GUIs, which were subsequently evaluated by physicians under realistic task conditions, combining subjective usability ratings with objective interaction data from eye-tracking. Figma^1^ was chosen as an established prototyping tool for early GUI design, allowing rapid visualization and feedback collection in the first cycle, while PyQt^2^ provided a high-fidelity implementation for functional evaluation under realistic task conditions in the second cycle.

### Application-specific GUIs

**Fig. 1:**
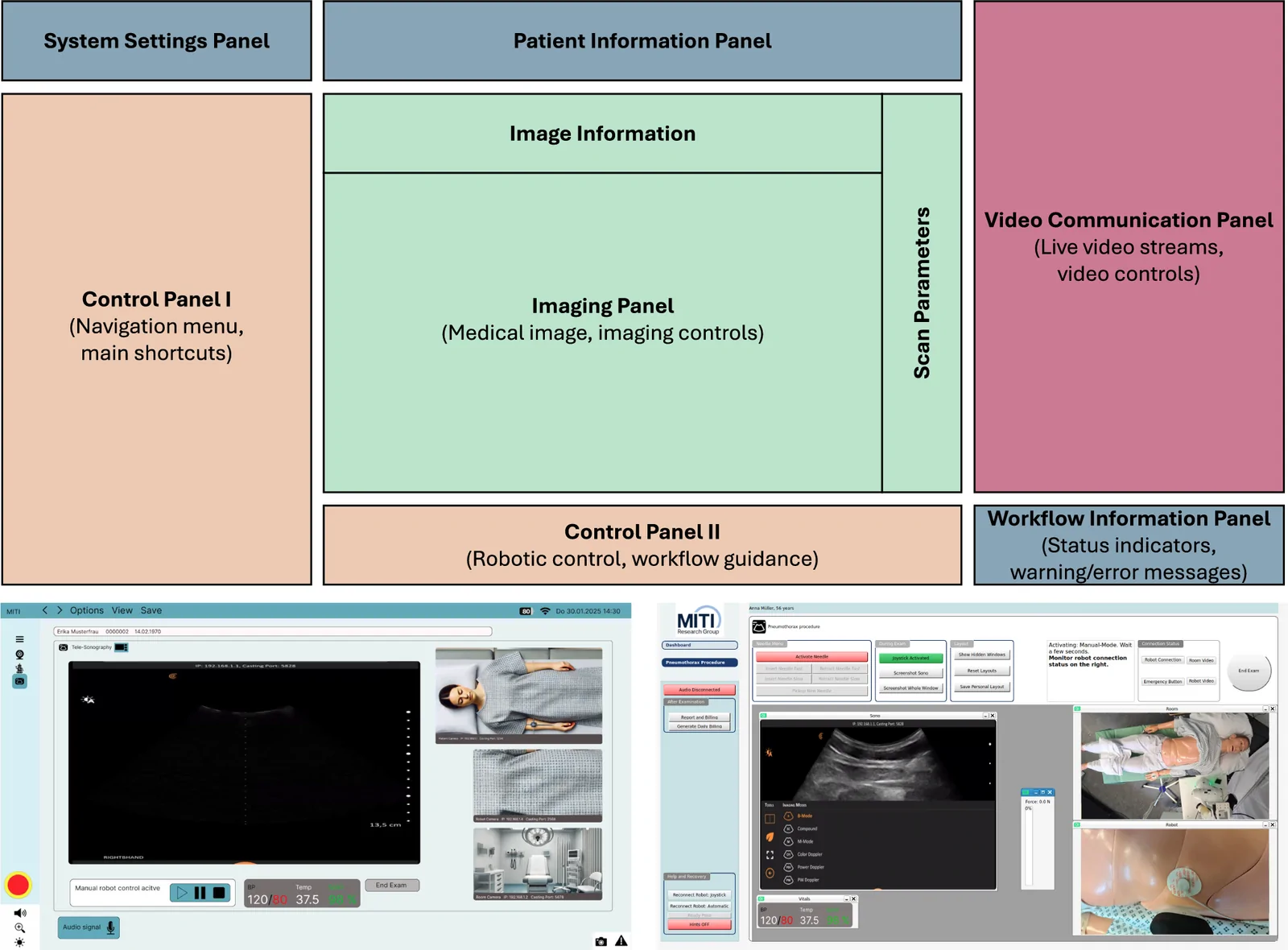
Generalized, modular GUI architecture concept. **Top:** Clustered layout of core elements of the examination interface. **Bottom left:** Static GUI prototype. **Bottom right:** Functional implementation for robotic tPTX assistance with workflow-specific modules arranged according to the generalized layout (note that *Control Panel II* was repositioned for this use case following user feedback)

Figure 1 shows an overview of a generalized GUI architecture for telemedical robotic systems, clustering all relevant information within core GUI areas. The overall arrangement follows a modular panel layout, where interface areas can adapt in size and visibility depending on the clinical workflow. To support efficient navigation, a unified navigation bar on the left-hand side ensures consistency across scenarios and aligns with design practices observed in hospital information systems. This layout concept can be adapted during the development phase of a new system, enabling the implementation of application-specific requirements and workflows while retaining higher-level structures and interaction paradigms.

The following section describes two exemplary use cases for which GUIs were designed based on this generalized layout:

- **Use case A (telesonography):** The first use case enables remote ultrasound diagnostics via a RUSS [46, 47]. For this, the GUI should provide clear feedback and incur minimal cognitive overhead while adapting to different examination modes. The clinical workflow is as follows: First, the remote physician selects an examination target in a live video stream of the patient. This target is transmitted to the robotic system, which subsequently plans and executes a trajectory to reach the selected point. Afterward, the robot switches to a manual teleoperation mode, enabling fine adjustments of the ultrasound probe position by the physician. This process is repeated until all points of interest have been examined.
- **Use case B (tPTX):** The second use case addresses robotic assistance for emergency needle decompression of suspected tension pneumothorax. As in telesonography, a remote physician remains in control and is supported by a GUI which is optimized for time-critical, safety-relevant decisions. The interventional workflow begins with the selection and installation of the decompression module on the robotic arm. Using live video and ultrasound feedback, the physician identifies the targeted thoracic region and confirms the presence of tPTX as well as a suitable intercostal space. The robotic system autonomously plans a safe approach trajectory, after which the physician performs fine positioning under teleoperation. Once the insertion site is confirmed, the robot executes the needle insertion along the planned axis, followed by controlled needle retraction while leaving the catheter in place. Decompression success is assessed via sensor feedback and patient monitoring, and the procedure can be repeated if necessary.

### Evaluation study design

**Fig. 2:**
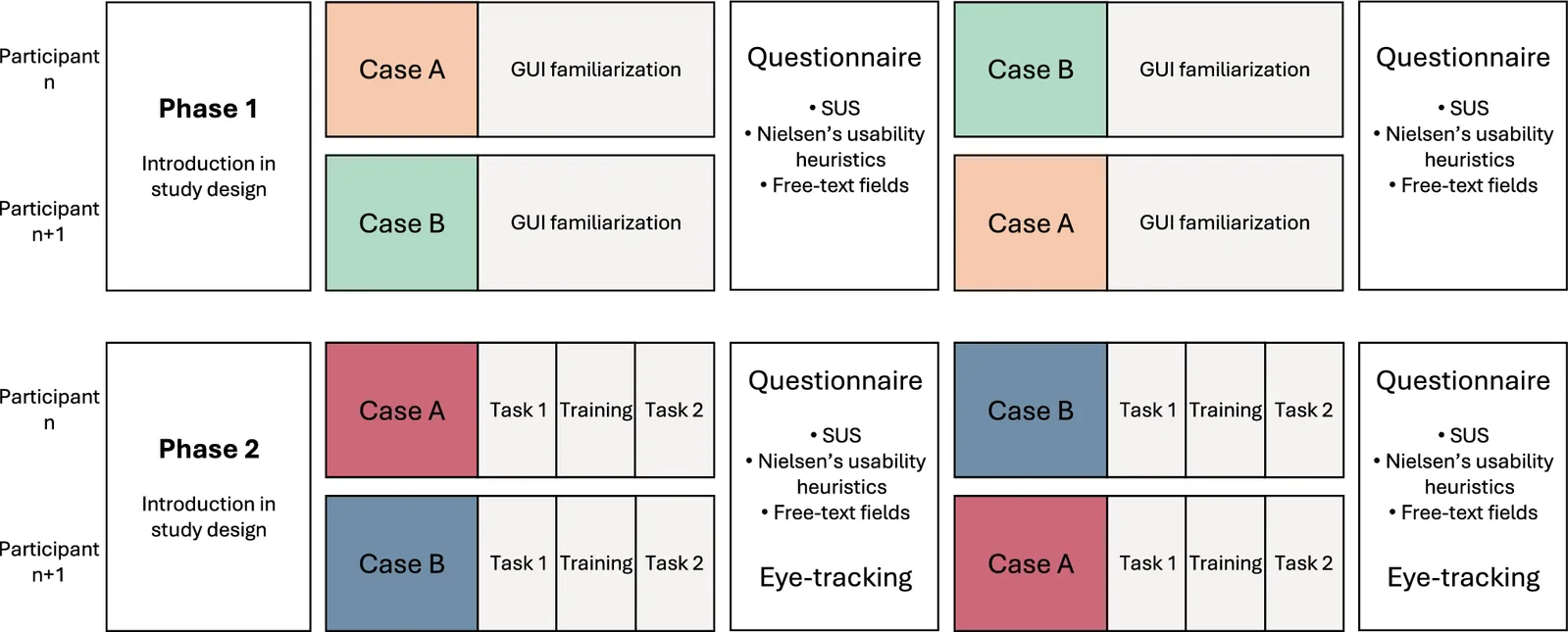
Graphical overview of the study design

A graphical summary of the study design is shown in Fig. 2. The study was conducted in two phases at the TUM University Hospital, with each phase corresponding to a UCD cycle. Participants interacted with a static GUI prototype in phase 1 and a functional GUI in phase 2. The evaluation was performed at a clinical desk setup, while the simulated remote patient site was located behind a partition wall. The setup included a RUSS comprised of a Panda robotic arm (*Franka Robotics GmbH*, Germany) with a custom end effector for each use case, multiple camera streams, and a patient mannequin. A study assistant was present throughout the study to ensure a standardized procedure and participant safety, and sessions were coordinated to minimize disruption to routine care. In phase 1, participants were tasked with performing the corresponding workflow for each use case as described above. In phase 2, participants completed a workflow-related set of tasks (e.g., "start a general examination with a patient of your choice"), followed by a short training session with exploratory interaction to reduce novelty effects, and finally another set of tasks. Before each task, participants were instructed that their ratings should refer specifically to the GUI and not to back-end functions, network delays, or robotic hardware performance. The order of use cases was randomized to minimize order effects. The study was reviewed and approved by the Ethics Committee of the Technical University of Munich. To comprehensively evaluate the usability of the developed GUIs, a mixed-methods approach was adopted, combining the following evaluation tools:

- **System usability:** SUS was chosen as a standardized and widely validated metric to quantify overall usability [45, 48]. Responses were rated on a 5-point Likert scale and subsequently converted into a total score ranging from 0 to 100 [45].
- **Heuristic evaluation:** To assess domain-independent aspects of GUI quality, Nielsen’s usability heuristics were reformulated into a structured questionnaire format [43, 44]. The resulting questionnaire items are listed in Fig. 4 as I1-12. Items were rated on a 5-point Likert scale and augmented with free-text fields, encouraging participants to specify design issues or desired improvements. These free-text responses were then coded against Nielsen’s categories and consolidated into thematic clusters. In phase 2, the heuristic items were supplemented with an additional question regarding the perceived training effect.
- **Eye-tracking:** Eye-tracking aimed to complement the questionnaires with objective evidence on visual attention patterns and interaction efficiency under realistic task conditions. Eye-tracking was applied only in phase 2, as the static prototype did not allow meaningful interaction besides page switching. Data were recorded using SMI Eye Tracking Glasses (*SensoMotoric Instruments GmbH*, Germany), with a three-point calibration before each session. Constant lighting conditions and a controlled study environment ensured comparability across participants. The eye-tracking metrics collected in the study included fixations (location and duration of gaze on predefined Areas of Interests (AOIs)) and saccades (eye movements between fixations). From these, the following derived measures were calculated: Dwell time (total time spent on an AOI), Time to First Fixation (TTFF) (time from stimulus onset until first fixation on the AOI), and fixation count (number of times an AOI was fixated). The AOIs used in this study corresponded to the workflow-specific GUI modules shown in Fig. 1 (e.g., ultrasound image module, help/recovery functions module, etc.).

## Results

### System usability

**Fig. 3:**
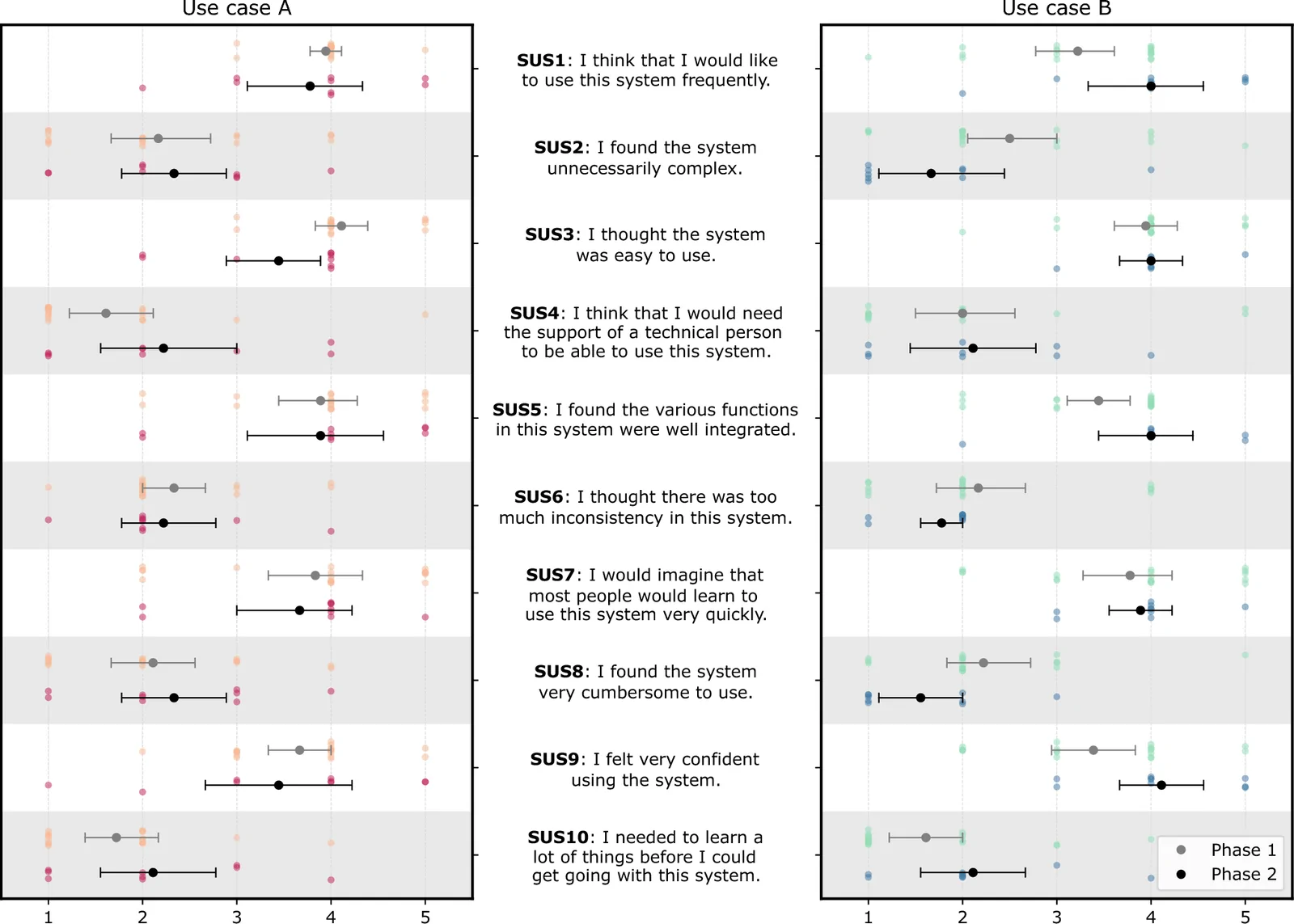
SUS item-level ratings for both use cases across evaluation phases. Dots represent individual participant responses on a 5-point Likert scale. Markers indicate mean values, and error bars denote standard deviations for phase 1 (n=18) and phase 2 (n=9)

The results of the SUS questionnaire for both use cases and evaluation phases are shown in Fig. 3. To compare perceived usability between phases, Welch’s independent samples t-tests (α=0.05) were conducted for each use case, as participants were not tracked across phases and sample sizes differed. Given the small and unequal sample sizes resulting from participant availability, the results should be interpreted as exploratory trends. For use case A (telesonography), SUS scores were slightly higher in phase 1 (73.75±13.01, n=18) than in phase 2 (67.50±20.50, n=9). This difference was not statistically significant (p=0.4213). In contrast, use case B (tPTX) showed an increase in SUS scores from the first evaluation round (68.19±16.40, n=18) to the second round (76.94±14.67, n=9), although this improvement did also not reach statistical significance (p=0.1776).

### Heuristic evaluation

**Fig. 4:**
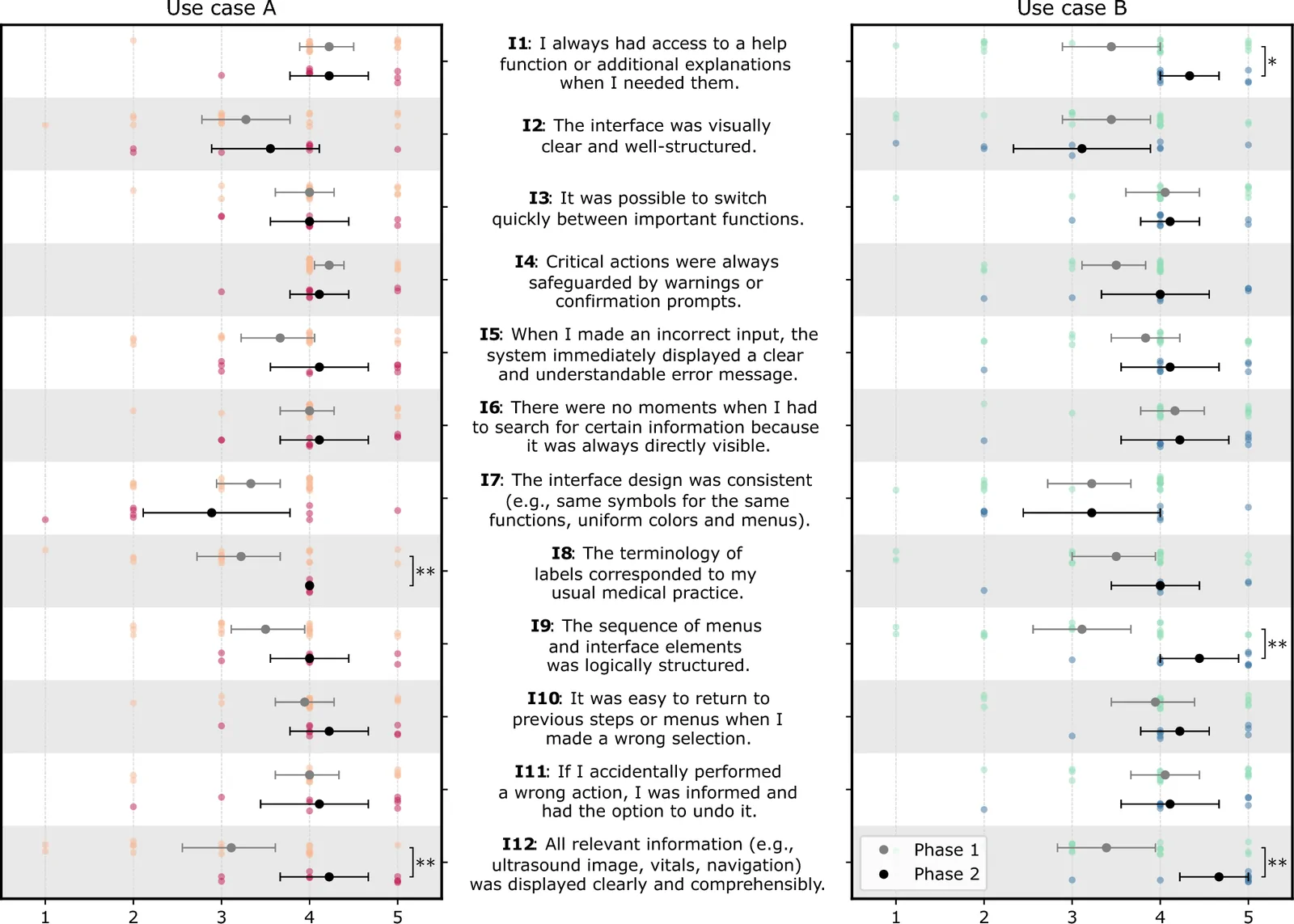
Item-level ratings of the heuristic evaluation for both use cases across evaluation phases. Dots represent individual participant responses on a 5-point Likert scale. Markers indicate mean values, and error bars denote 95% confidence intervals for phase 1 (n=18) and phase 2 (n=9), and asterisks denote statistically significant item-wise comparisons (α=0.05)

The results of the heuristic evaluation for both use cases and evaluation phases are shown in Fig. 4. To assess changes between the static prototype and the functional implementation, item-wise and aggregated heuristic scores were compared using Welch’s independent samples t-tests (α=0.05). Again, given the small and unequal sample sizes, these results should be interpreted as exploratory trends. For use case A, item-level scores related to the matching of terminology to clinical routines (I8) and clear visibility of information (I12) showed significant improvements across phases (p=0.0063 and p=0.0099, respectively). The aggregated heuristic rating also increased from phase 1 (3.71±0.44, n=18) to phase 2 (3.96±0.56, n=9), although no statistically significant difference was observed between evaluation phases (p=0.2526). For use case B, item-level ratings related to help functions (I1), menu and interface logic (I9), and clear visibility of information (I12) displayed significant improvements across phases (p=0.0172, p=0.0017 and p=0.0019, respectively). Aggregated heuristic scores showed a tendency toward improvement from phase 1 (3.64±0.61, n=18) to phase 2 (4.05±0.57, n=9), although this difference did not reach statistical significance (p=0.1067). After the training phase, participants reported improved skills with a mean score of 4.78±0.42 (n=9) in both cases.

### Eye-tracking

**Fig. 5:**
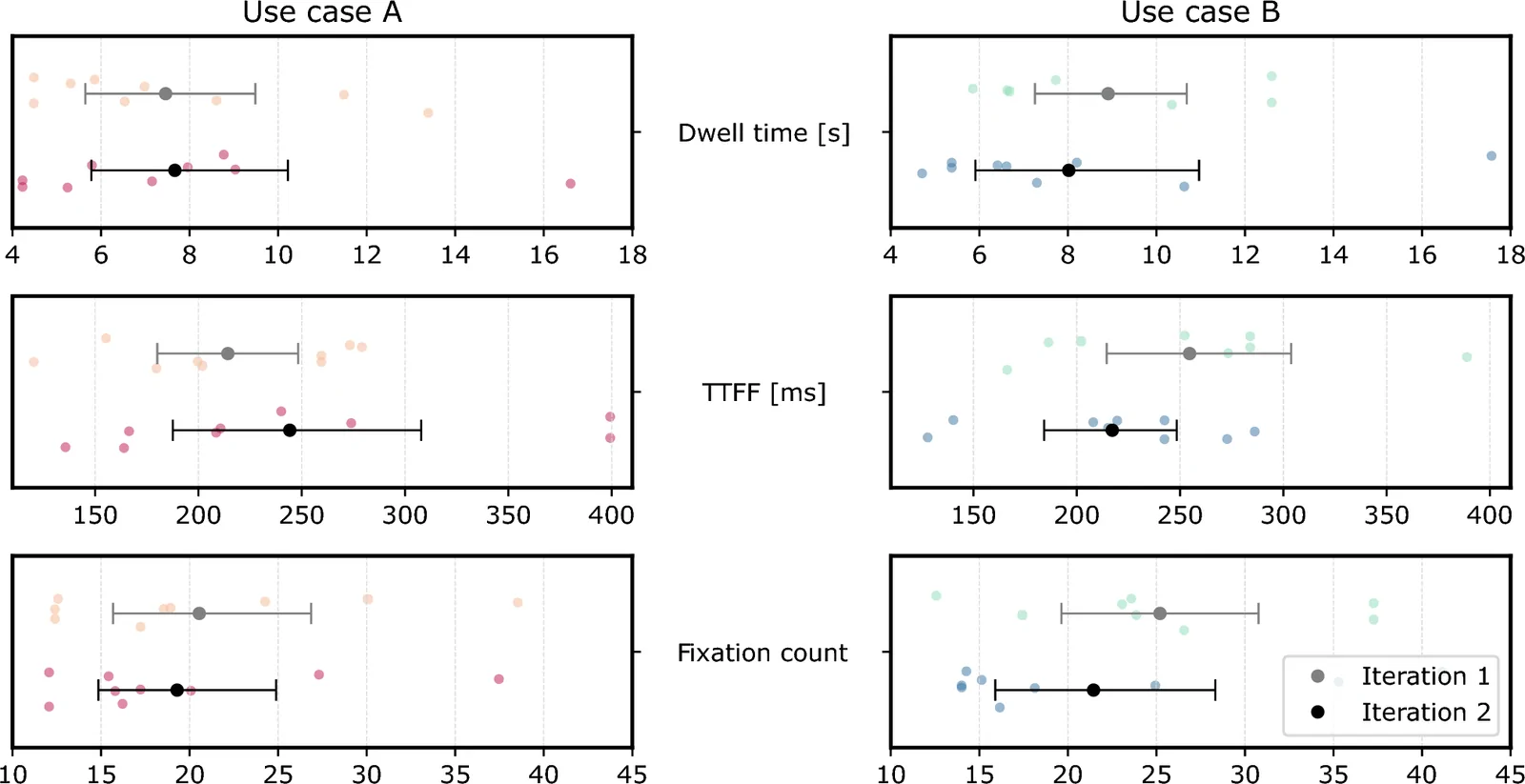
Eye-tracking metrics recorded during phase 2 for both use cases. Results are shown separately for initial task execution (iteration 1, top) and post-training task execution (iteration 2, bottom). Dots represent individual participant values. Markers indicate mean values and error bars denote standard deviations (n=9)

Eye-tracking data were collected in two consecutive iterations: an initial task execution (iteration 1), followed by a training phase, and a second task execution (iteration 2). This design enabled the analysis of visual attention and interaction behavior before and after familiarization with the system. Figure 5 summarizes the distributions of the analyzed eye-tracking metrics for both use cases and task iterations. For use case A, dwell time remained largely stable, changing from 7.46±3.14 s in iteration 1 to 7.67±3.81 s in iteration 2. First fixation occurred slightly later, with TTFF increasing from 214.24±56.63 ms to 244.21±97.26 ms. Fixation count decreased from 20.55±8.96 to 19.30±8.22. For use case B, dwell time decreased from 8.91±2.68 s in iteration 1 to 8.02±4.00 s in iteration 2. In contrast to use case A, first fixation occurred earlier in iteration 2, with TTFF decreasing from 254.63±71.04 ms to 217.20±53.76 ms. Fixation count decreased from 25.20±8.63 to 21.45±10.20. Across both use cases, iteration 2 was characterized by lower fixation counts, suggesting more focused visual attention. Changes in dwell time and first fixation latency were more use case dependent, indicating that familiarization affected visual behavior differently depending on task structure and information layout.

## Discussion

Evidence indicates that a User-centered GUI development process with multiple evaluation rounds can improve the usability of telemedical robotic systems for medical professionals. For the tPTX use case, SUS scores increased across evaluation phases, which is consistent with design changes to integrate user feedback. Although SUS scores decreased slightly for the telesonography use case, this pattern is plausible since the functional implementation introduced additional interaction demands and exposed real system states that the static prototype did not reveal. Across both iterations and both use cases, SUS scores consistently indicated a “Good” level of perceived usability (according to Bangor et al.) [48].

Heuristic ratings and free-text responses reflected usability improvements due to design changes between phases. After the first evaluation, participants highlighted workflow alignment needs, such as matching of medical terminology to clinical routines, visibility of relevant information and access to help functions, as key areas for improvement. In addition, participants emphasized that the interface should be more functionally integrated, with less hierarchical structuring and more personalization options to better support their workflows. After the second evaluation cycle, participants commended improvements in these aspects, but also noted persistent information gaps. Recurrent requests included richer error feedback and concerns about system responsiveness. In use case B specifically, clinicians emphasized the need for more needle-related cues, such as penetration depth and needle visualization during application, even when global system usability remained good. A detailed summary of the initial requirements captured in the analysis step of the first UCD cycle, as well as the main requirements derived from user feedback in the analysis step of the second UCD cycle, is presented in Online Resource 1.

The eye-tracking data gathered in phase 2 show an increased efficiency across different tasks, iterations, and use cases, indicating a measurable training and familiarization effect. Most participants exhibited similar dwell times and slightly lower fixation counts in the second run of the same GUI, showing that repeated exposure reduced search effort. For use case B, TTFF correspondingly decreased post-training, possibly indicating a stronger training effect for this scenario. The heuristic evaluation supports this finding, as the training item received high agreement for both use cases. Furthermore, groups that had already completed tasks with one GUI performed faster with the other GUI than groups encountering that interface for the first time. This pattern indicates a cross-scenario learning transfer leading to more efficient gaze behavior and shorter search times, pointing to a good overall consistency across the two use cases. It further highlights the benefits of a modular GUI architecture, preserving high-level structures and interaction concepts across implementations. This finding is particularly interesting in the context of service-oriented or microservice architectures for medical applications, which could benefit from adaptable, yet structurally consistent and familiar GUIs to enable clinicians to perform tasks without incurring a high learning burden [49].

The eye-tracking data added behavioral evidence which was especially valuable given the small sample size in phase 2, since it increased confidence in questionnaire trends and helped to analyze participant-level outliers via triangulation. As an example, for one participant, long search times and a high fixation count support their low subjective rating (SUS <50). For another participant with a very low rating for use case A (SUS <30) and an average rating for use case B, the eye-tracking results do not produce a corresponding pattern indicating confusion or pronounced search effort. Instead, search times improved from the first to the second run in both use cases.

Finally, combining objective eye-tracking and subjective questionnaires provided complementary insights, which helped to reveal additional design issues not captured by questionnaires. Although both GUI iterations displayed live system states in the status area and highlighted transitions between autonomous and manual control via explicit prompts in the info label, gaze behavior showed no consistent dwell time pattern across GUIs or runs, suggesting that control mode changes were not sufficiently communicated in the intended area. Two design implications follow from this finding. First, feedback related to mode changes should be made more prominent, e.g., through stronger visual transitions, clearer affordances, and tighter synchronization between the status area and the info label. Second, dependence on the status area could be reduced by reinforcing task-embedded cues, such as characteristic force patterns after a successful transition. The observed behavior also showed that users would sometimes resort to exploratory actions (e.g., repeated joystick movement) to verify a control handover, underscoring the need for clearer confirmations. These details complement the SUS scores and heuristic ratings and explain why some users still experienced search effort or uncertainty in otherwise well-rated GUIs.

## Conclusion

The results of the study confirm the benefits of UCD methods for the development of GUIs for telemedical robotic systems. To enable the translation of usability findings into design and functional adjustments, the GUI should be treated as an integral part of telemedical robotic systems and should be developed in close coordination with underlying functional components. A modular architecture can facilitate the transfer of training effects across implementations and clinical use cases by preserving high-level structures and interaction concepts. Naturally, GUI development should be supported by risk-based safety assessment and documentation to align with regulatory requirements.

**Supplementary information:** Online Resource 1 (Tables with initial requirements captured in the analysis step of the first UCD cycle and main requirements derived from user feedback in the analysis step of the second UCD cycle.)

## Supplementary Information

Below is the link to the electronic supplementary material.

Supplementary file 1 (pdf 112 KB)

## Footnotes

1 https://www.figma.com/.

2 https://www.riverbankcomputing.com/software/pyqt.
