# Supplementary material for: User-centered design of graphical user interfaces for telemedical robotic systems: development and usability study
Source: Int J Comput Assist Radiol Surg. 2026 Jun 15;21(10):2087–97. doi: 10.1007/s11548-026-03701-4 (PMC13723647; doi:10.1007/s11548-026-03701-4)
Supplement: Supplementary file 1 [file 11548_2026_3701_MOESM1_ESM.pdf]

International Journal of Computer Assisted Radiology and Surgery

User-centered Design of Graphical User Interfaces for Telemedical Robotic Systems: Development and Usability Study – Online Resource 1

Sven Kolb[1,2*], Marie Kautt[1,3], Lars Wagner[1,2], Simon Saurbier[3], Sven Matthiesen[3], Dirk Wilhelm[1,2,4], Carolin Müller[5]

[1*]Technical University of Munich, School of Medicine and Health, TUM University Hospital, Research Group MITI, Munich, Germany.
[2]Technical University of Munich, Munich Institute of Robotics and Machine Intelligence (MIRMI), Munich, Germany.
[3]Karlsruhe Institute of Technology (KIT), Institute of Product Engineering (IPEK), Karlsruhe, Germany.
[4]Technical University of Munich, School of Medicine and Health, TUM University Hospital, Department of Surgery, Munich, Germany.
[5]Technical University of Munich, School of Medicine and Health, TUM University Hospital, Department of Trauma Surgery, Munich, Germany.

*Corresponding author(s). E-mail(s): sven.kolb@tum.de;

Requirements

Table 1: Initial requirements captured in the analysis step of the first User-centered Design (UCD) cycle.

**Use case A (telesonography)**		**Use case B (tension pneumothorax)**	
R1.1A	Select examination mode	R1.1B	Select end effector/needle
R1.2A	Activate autonomous motion	R1.2B	Trigger needle insertion/retraction
R1.3A	Display vital parameters	R1.3B	Display robot visualization
**Both use cases**			
R1.4	Display video streams	R1.12	Multiple interface layers
R1.5	Display ultrasound image stream	R1.13	Customizable views
R1.6	Display contact force	R1.14	Provide direct hover feedback
R1.7	Display procedure-specific controls	R1.15	Navigation bar on the left side
R1.8	Display system feedback	R1.16	Highlight current workflow step
R1.9	Activate manual control	R1.17	Self-descriptive labels/icons
R1.10	Emergency stop button always visible	R1.18	Double-click to undo/reverse action
R1.11	End procedure button always visible	R1.19	Consistent design and color scheme

Table 2: Main requirements derived from user feedback in the analysis step of the second UCD cycle.

**Use case A (telesonography)**		**Use case B (tension pneumothorax)**	
R2.1A	Shortcut to switch examination mode	R2.1B	Display vital parameters
R2.2A	Clearer visual distinction between modes	R2.2B	Confirmation prompt before needle insertion
R2.3A	Display room video stream across all layers	R2.3B	Provide post-puncture feedback
R2.4A	Medical terminology should match clinical routines	R2.4B	Display additional video stream for better overview
**Both use cases**			
R2.5	Maintain consistent window layouts	R2.11	Provide a clear help button
R2.6	Keep feedback/error messages concise	R2.12	Provide documentation templates
R2.7	Display warnings more prominently	R2.13	Enable direct switching between dashboard and examination/procedure
R2.8	Provide an option to display all views simultaneously	R2.14	Reduce hierarchical structures and improve customization
R2.9	Remove brightness/zoom controls	R2.15	Only display necessary technical information
R2.10	Display system status across all layers	R2.16	Provide a step-by-step assistance mode
